# Application of dicentric chromosome assay for evaluation of radioprotective effect

**DOI:** 10.1093/biomethods/bpaf058

**Published:** 2025-08-05

**Authors:** Marcela Milanová, Vojtěch Chmil, Aleš Tichý, Lenka Lecová

**Affiliations:** Department of Radiobiology, Military Faculty of Medicine, University of Defence, Třebešská 1575, Hradec Králové, 500 02, Czech Republic; Department of Radiobiology, Military Faculty of Medicine, University of Defence, Třebešská 1575, Hradec Králové, 500 02, Czech Republic; Department of Radiobiology, Military Faculty of Medicine, University of Defence, Třebešská 1575, Hradec Králové, 500 02, Czech Republic; Department of Radiobiology, Military Faculty of Medicine, University of Defence, Třebešská 1575, Hradec Králové, 500 02, Czech Republic; Department of Molecular Pathology and Biology, Military Faculty of Medicine, University of Defence, Třebešská 1575, Hradec Králové, 500 01, Czech Republic

**Keywords:** amifostine, radioprotective agent, cytogenetics, ionizing radiation, chromosome aberrations

## Abstract

The dicentric chromosome assay is a well-established biodosimetric method used to assess absorbed ionizing radiation doses by detecting dicentric chromosomal aberrations. Here, we present a detailed, reproducible protocol for applying the dicentric chromosome assay for *in vitro* evaluation of radioprotective agents, including novel piperazine derivatives compared with amifostine and its active metabolite WR-1065. The protocol covers all key steps—blood sample preparation, *in vitro* irradiation, lymphocyte culture, metaphase preparation, and scoring of dicentric chromosomes. It highlights critical stages that affect data quality and reproducibility. Integrating manual scoring with automated analysis using the Metafer system ensures accurate and efficient assessment. Thus, this protocol bridges the fields of biological dosimetry and preclinical screening of radioprotective agents, providing a reliable framework for emergency radiation dose estimation and the development of new radiation medical countermeasures.

## Introduction

The number of patients exposed to ionizing radiation (IR) for both diagnostic and therapeutic purposes is increasing worldwide, and drugs that could be applied to prevent or mitigate the negative consequences of radiation are essential [1–4]. In addition, due to the growing risk of nuclear accidents and the potential deployment of nuclear weapons, the development of medical radiation countermeasures is becoming a key area of security and defense research [5]. Radioprotective agents can significantly minimize the long-term health risks of radiation exposure, such as increased cancer risk, and the effects of acute radiation syndrome [6]. One approach to evaluating a radioprotective agent’s effectiveness is the dicentric chromosome assay (DCA) [7].

The DCA is a specific and sensitive technique for estimating exposure to IR, even at low doses. This assay detects specific chromosomal aberrations, whose formation correlates directly with the absorbed radiation dose [8, 9]. Its reliability and accuracy have been confirmed in international comparative studies, and it is widely used in acute radiological events where the rapid and efficient triage of victims is needed [10, 11].

We employed the DCA to evaluate the efficacy of novel in-house synthesized piperazine derivatives compared with amifostine and its active metabolite WR-1065, which is still considered a benchmark in radioprotection. We assessed how these agents mitigated radiation-induced damage by analyzing the frequency of dicentric chromosomes (DCs) in exposed peripheral blood lymphocytes. The results demonstrated that the DCA is a valuable tool for evaluating radioprotective efficacy, providing critical data for appropriate medical response and developing new treatments [7, 12].

The innovative aspect of this method lies in its comprehensive application to measure radiation exposure and systematically assess the efficacy of radioprotective compounds. This research bridges the gap between traditional biological dosimetry and therapeutic radioprotection, offering dual utility in emergency radiation exposure assessment and proactive development of protective medical interventions.

This detailed protocol provides a step-by-step procedure, highlighting critical steps and notes that can significantly impact the entire analysis, potentially leading to poor sample quality and unsuccessful study outcomes. Moreover, the comprehensive description of the setup and evaluation process using the automated scanning system Metafer enhances the reproducibility and accuracy of the assay. As such, this protocol supports further scientific exploration and strengthens its practical utility in emergency radiation response and the ongoing development of effective radioprotective strategies. This protocol article offers an in-depth description of the experimental method outlined in our recently published research study [7].

## Experimental design

The samples were collected from 12 healthy male donors (8 mL of blood from each donor) who do not regularly take medication. Human peripheral blood samples were divided into three groups: (i) Control group (no radiation, no treatment); (ii) Irradiated-only group (only radiation); (iii) Treatment group (radiation with previous treatment). Each group was tested in three replicates (blood from three different donors) to balance feasibility and reliability. Before irradiation, the treated groups received the tested compound at optimized concentrations, which were determined experimentally on lymphocytes via cytotoxicity assays [7]. After radiation exposure, the lymphocytes were cultured with necessary supplements and specific mitogens and then processed for DCA. The DCA preparation involves several stages characterized by specific reagents. The results were statistically evaluated to determine the reduction in DCs, expressed as a radioprotection factor (RF). This approach provides insights into the efficacy of tested compounds in protecting cells against radiation-induced chromosome damage.

## General solutions and reagents

- Amifostine (Sigma-Aldrich, St Louis, MO, USA; Cat. No.: 1019406)
- Cell Culture Medium: RPMI 1640 Medium (ATCC modification) (Gibco™, Thermo Fisher Scientific, Waltham, MA, USA; Cat. No.: A1049101)
- Dimethyl Sulfoxide (Sigma-Aldrich, St Louis, MO, USA; Cat. No.: D5879)
- Deionized Water (Sigma-Aldrich, St Louis, MO, USA; Cat. No.: 38796)
- Dulbecco’s Phosphate Buffered Saline (Sigma-Aldrich, St Louis, MO, USA; Cat. No.: D8662)
- Fetal Bovine Serum, heat-inactivated, qualified, Canada, One Shot™ format (Gibco™, Thermo Fisher Scientific, Waltham, MA, USA; Cat. No.: A3840302)
- Giemsa-Romanowski Solution (Penta, Prague, Czech Republic; Cat. No.: 14460-11000)
- Glacial Acetic Acid 99.8% p.a. (Penta, Prague, Czech Republic; Cat. No.: 19990-11000)
- Gurr Buffer Tablets (Gibco™, Thermo Fisher Scientific, Waltham, MA, USA; Cat. No.: 10582013)
- Immersion Oil (Carl Roth, Karlsruhe, Germany; Cat. No.: R.X899.2)
- KaryoMAX™ Colcemid™ Solution in PBS (10 μg/mL) (Gibco™, Thermo Fisher Scientific, Waltham, MA, USA; Cat. No.: 15212012)
- Methanol p.a. (Penta, Prague, Czech Republic; Cat. No.: 21210-11000)
- Penicillin-Streptomycin Solution with 10 000 U of penicillin and 10 mg of streptomycin/mL (Sigma-Aldrich, St Louis, MO, USA; Cat. No.: P4333).
- Phytohaemagglutinin, M form (Gibco™, Thermo Fisher Scientific, Waltham, MA, USA; Cat. No.: 10576015)
- Potassium Chloride Solution 0.075 M (Gibco™, Thermo Fisher Scientific, Waltham, MA, USA; Cat. No.: 10575090)
- WR-1065 ≥98% (HPLC) (Sigma-Aldrich, St Louis, MO, USA; Cat. No.: W2020)

## Glassware and plasticware

- BD Vacutainer^®^ Heparin Tubes 6 mL (Becton, Dickinson and Company, Franklin Lakes, NJ, USA; Cat. No.: 367886)
- Costar^®^ Serological Pipettes (5, 10, 50 mL) (Sigma-Aldrich, St Louis, MO, USA; Cat. No.: CLS4487, CLS4488, CLS4490)
- Eppendorf Conical Tubes 15 mL (Eppendorf, Hamburg, Germany; Cat. No.: 0030122151)
- Eppendorf Safe-Lock Tubes 1.5 mL (Eppendorf, Hamburg, Germany; Cat. No.: 0030123611)
- Microscope slides 26 × 76 mm (P-Lab, Prague, Czech Republic; Cat. No.: M100200.1)
- Microscope slide box KARTELL (P-Lab, Prague, Czech Republic; Cat. No.: K000278)
- Plastic Pasteur Pipettes PE 3 mL (P-Lab, Prague, Czech Republic; Cat. No.: K000334)
- Staining Jar (P-Lab, Prague, Czech Republic; Cat. No.: H999355)
- Tissue Culture Flasks 25 cm^2^ (TPP™, Trasadingen, Switzerland; Cat. No.: 90026)

## Laboratory equipment

- ^60^Co irradiator Chisostat^®^ (Chirana, Prague, Czech Republic)
- Accu-jet^®^ pro Pipette Controller (Brand^®^, Sigma-Aldrich, St Louis, MO, USA; Cat. No.: Z637688)
- Automated Scanning and Imaging Platform Metafer Version 4.3.8 (MetaSystems Hard & Software GmbH, Altlußheim, Germany) including a microscope, a high-resolution CCD (charged-coupled device) camera connected to a capturing device for real-time image digitization, a mobile scanning platform, a trackball for manual movements, a fully equipped computer, and a hard drive with sufficient capacity for archiving.
- Centrifuge LMC-3000 (Biosan Ltd, Riga, Latvia; Cat. No.: BS-010208-AAA)
- CO_2_ incubator (PHCbi, Tokyo, Japan; Cat. No.: model MCO-170AICUV-PE)
- Combined refrigerator with freezer (−20°C) (Bosch, Renningen, Germany; model KGN36VW30)
- Eppendorf Research plus^®^ Pipettes (Eppendorf, Hamburg, Germany; Cat. No.: 3123000942)
- JB Academy Unstirred Water Bath (Grant Instruments Ltd, Royston, UK; Cat. No.: JBA12)
- Microbiological Safety Cabinet 1.2 Safeflow (Bioair, Siziano, Italy; model 1.2 Safeflow)
- Optical microscope (Olympus, Tokyo, Japan; model BX51)
- pH meter FiveGo F2 (Mettler Toledo, Prague, Czech Republic; Cat. No.: LE 438-IP67)
- PHOENIX Vacuum Aspiration System VAS-10 (Phoenix Instrument, Garbsen, Germany; Cat. No.: VAS-10)
- Roller Mixer RM 5/200 (Glaswarenfabrik Karl Hecht GmbH & Co KG, Sondheim, Germany)
- Vortex Minishaker MS2 (IKA-Werke GmbH & Co. KG, Staufen, Germany; model MS2)

## Software

- Metafer 4, version 4.3.8 (MetaSystems Hard & Software GmbH, Altlußheim, Germany)
- GraphPad Prism 8.4.3 (GraphPad Software Inc., Boston, MA, USA)

## Methods

### Reagent setup

#### Radioprotective substances

1. Prepare a 100 mM stock solution of amifostine by dissolving the required amount in Dulbecco’s phosphate-buffered saline. Amifostine can be stored in solution for 1 month at −20°C or up to 6 months at −80°C.
2. Prepare a 100 mM stock solution of WR-1065 by dissolving the required amount in dimethyl sulfoxide. The solution can be stored for 1 month at −20°C or up to 2 years at −80°C.
3. Prepare a 100 mM stock solution of piperazine derivatives by dissolving the required amount in dimethyl sulfoxide.
4. Prepare 1 mM working solutions of tested compounds by diluting respective stock solutions in RPMI 1640 medium. Always prepare fresh working solutions before each experiment.

#### Cell culture medium

1. Prepare the appropriate volume of RPMI 1640 culture medium (containing L-glutamine and glucose) supplemented with 10% fetal bovine serum (FBS), 2% phytohaemagglutinin (PHA) M form, and 1% ATB (penicillin/streptomycin). See Problem 1 for more information.
2. Always prepare fresh medium before each experiment. Store all aliquots of culture medium supplements at −20°C.
**Critical:** Do not thaw FBS and PHA in a water bath. Let them thaw at room temperature. Avoid repeated freeze–thaw cycles of PHA aliquots.
**Note:** The form and type of PHA are optional. Both PHA L and M can be used to maintain optimal lymphocyte stimulation. If using the lyophilized powder of PHA, first prepare a solution. In any case, achieving a final concentration of 20 μg/mL in the blood culture is necessary.

#### Cell fixation solution

1. Mix methanol and glacial acetic acid in a 3:1 ratio to prepare the fixative solution. See Problem 2 for additional information.
2. Always prepare a fresh fixative solution and cool it to −20°C before the fixation procedure.

#### Staining solution

1. Dissolve Gurr buffer tablets in the required volume of deionized water (one tablet per 100 mL) and check the pH of the solution (the solution should have a pH of 6.8). Gurr buffer solution can be stored for up to 1 month at 2–8°C.
2. Always prepare fresh 5% staining solution by adding 5 mL of filtered Giemsa-Romanowski stock solution to 95 mL Gurr buffer solution.

### Sample preparation

1. Collect peripheral blood from healthy donors into Li-heparin collection tubes and mix gently by rotating on a roller until processing.
**Critical:** Avoid using other anticoagulants such as ethylenediaminetetraacetic acid (EDTA) or sodium citrate (Na-citrate). Since calcium (Ca^2+^) is essential for lymphocyte stimulation by PHA, EDTA or Na-citrate, as chelating agents, may reduce cell division and proliferation in culture.
**Critical:** Avoid temperature changes. Keep blood samples at a temperature of 18–24°C until processing. Avoid cooling or excessive temperatures.
2. Divide the blood sample into three groups: 1 mL aliquots for the control group (non-irradiated, non-treated sample), 1 mL aliquots for the irradiated-only group (irradiated, non-treated sample), and 1 mL aliquots per substance for the treatment group (treated and irradiated sample).
3. Treat the samples with working solutions of tested compounds to the required final concentration.
4. Place the samples in an incubator with 5% CO_2_ and 100% relative humidity at 37°C for 30 min.
5. After 30 min of incubation, expose the treatment and irradiated-only groups to a single dose of 3 Gy of gamma radiation at a dose rate of 0.6 Gy·min^−1^ at room temperature using the ^60^Co irradiator. An additional sham-irradiated control group is subjected to the same protocol to account for handling stress.
6. Immediately after irradiation, return the samples to an incubator with 5% CO_2_ and 100% relative humidity at 37°C for 30 min.

### Cultivation

**Note:** Sample preparation and cell culture procedures must be performed under aseptic conditions inside a properly maintained and certified biosafety cabinet.

1. Add 8.2 mL of supplemented RPMI 1640 culture medium to T25 cell cultivation flasks and 800 μL of well-mixed whole blood (alternatively, 5 mL of medium and 500 μL of blood).
2. Mix thoroughly (do not shake) and incubate at 37°C, 5% CO_2_, and 100% relative humidity for 24 h.
3. Add colcemid to the culture to reach a final concentration of 0.074 μg/mL, mix gently, and incubate for 24 h. Thus, the total incubation time of the culture is 48 h. See Problem 3 for details.
**Optional:** Lymphocytes can be incubated for a standard period of 46–52 h. It is possible to add colcemid 2–3 h before terminating the culture, but this approach may require a higher final concentration of colcemid.
**Critical:** Colcemid’s final concentration and exposure time are crucial for proper metaphase arrest. Colcemid is usually added at a 0.05–0.1 μg/mL concentration. An insufficient concentration of colcemid decreases the yield of metaphases. Conversely, an excessive concentration or early addition leads to toxicity and undue cell contraction. The correct timing and adequate colcemid concentration in this protocol result in an optimal and sufficient number of high-quality M1 metaphases [8].

### Hypotonic solution treatment

1. After incubation, transfer the blood culture to a 15 mL tube and centrifuge at 600 × *g* for 3 minutes.
2. Remove most supernatant and thoroughly resuspend the pellet in the remaining volume.
3. Slowly add 0.075 M KCl solution (prewarmed to 37°C) using a Pasteur pipette, until the total volume reaches 10 mL. Mix thoroughly.
**Critical:** It is essential to resuspend the cells homogenously and to add the hypotonic solution slowly and consistently. Incorrect execution of this step may lead to irregular cell swelling, resulting in a low number of high-quality metaphases.
4. Incubate for 20 min at room temperature.
**Optional:** To save time, the incubation can be shortened to 10 min if held in an incubator at 37°C, 5% CO_2_, and 100% relative humidity. Optimizing this step for your laboratory conditions is highly recommended.

### Fixation

**Note:** Throughout the fixation process, it is essential not to aspirate the entire volume of supernatant/fixative after each centrifugation, but to retain a small amount above the cell pellet to prevent sudden evaporation of the fixative and drying of the sample. Before adding fresh ice-cold fixative, ensure the cell pellet is well resuspended, and add the fixative slowly while stirring to ensure that the cells are evenly dispersed and do not form clumps. See Problem 2 for troubleshooting information for the fixation process.

1. After incubation with KCl, centrifuge at 600 × *g* for 3 min at room temperature. Carefully aspirate the supernatant using an aspirator or a Pasteur pipette and thoroughly resuspend the pellet.
2. Slowly add a small volume (1 mL) of freshly prepared ice-cold fixative solution drop by drop. Mix thoroughly and add the fixative solution to a total volume of 8 mL while stirring continuously.
3. Centrifuge at 600 × *g* for 3 min at room temperature. Carefully aspirate the supernatant and thoroughly resuspend the pellet.
**Pause:** At this point, once fixed, cells can be stored briefly at 4°C.
4. Repeat steps 2 and 3 twice (or more if necessary) until the supernatant is clear and transparent, without coloration and/or opacity. After the last centrifugation, carefully aspirate the supernatant, leaving about 0.3 mL above the pellet, and thoroughly resuspend the pellet. See Problem 3 for details.
**Pause:** The cells fixed in this manner can be stored at 4°C for no longer than 24 h. Long-term storage is possible at −20°C.

### Slides preparation and staining

**Critical:** Degreased, clean, wet, and well-chilled microscope slides are required. Ensure the ambient temperature and humidity are constant during slide preparation and avoid ventilation. The humidity and temperature of the slides and the ambient conditions may affect the final quality of the slides.

**Figure 1.**
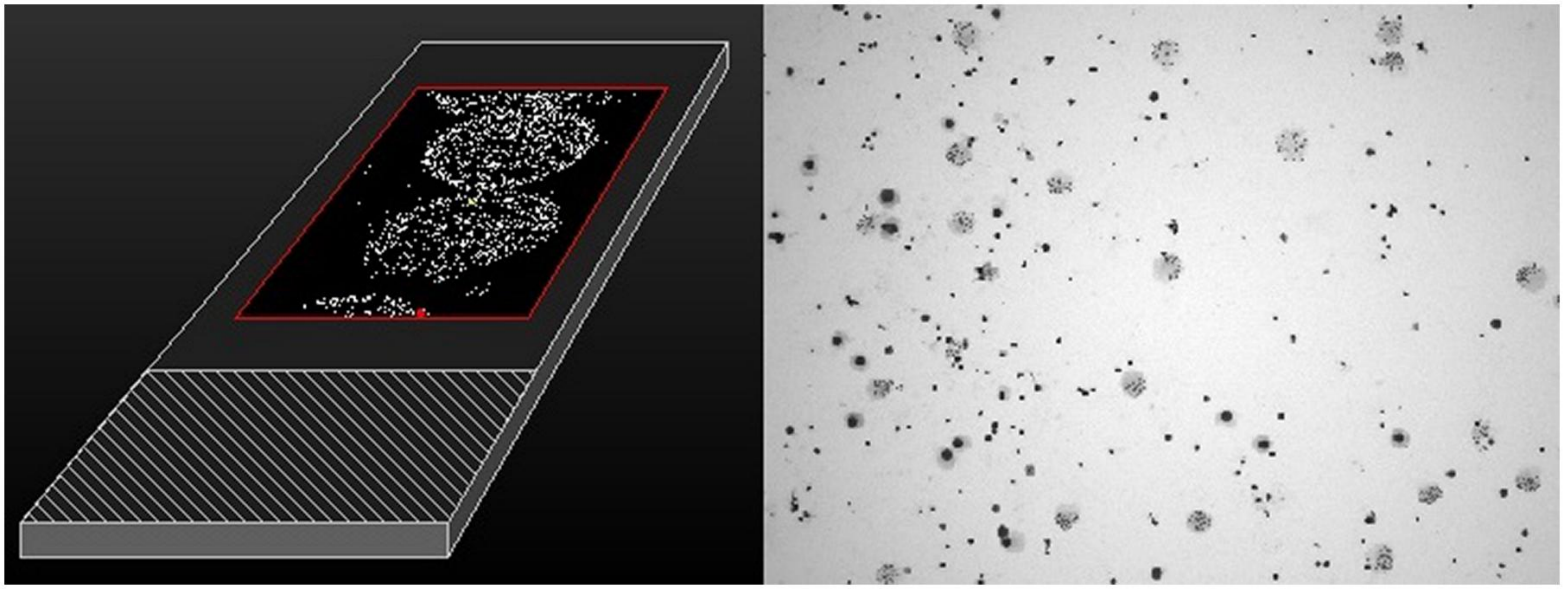
Optimal dripping position on the microscope slide for automated scanning (left) with the optimal spread and number of metaphases (right).

1. Degrease and clean microscope slides by immersing them in methanol and rinsing with deionized water. Keep the slides immersed in a container of deionized water with a drop of methanol in the fridge or freezer until use.
2. Mix the suspension well before dropping.
3. Remove the slide from the container, hold it by the edges in a horizontal position, and drop 2–3 drops of suspension (approximately 30 μl) with a pipette from a height of approximately 40 cm, sufficient to ensure the release of the nuclear content.
4. Check the overall density of metaphase cells and prepare two or more slides per sample if necessary.
**Note:** Drop the suspension along the midline of the slide for optimal spreading and to ensure the capture of a sufficient number of metaphases (Fig. 1).
5. Place the slides on a paper pad or towel and let them air dry for approximately 1 hour (as long as the slide is visibly dry).
6. Dip the slides into the fresh 5% staining solution for 5–8 min.
7. Rinse excess stain by holding the slide at an angle and squirting a gentle, indirect stream of cold water onto the slide, allowing it to run down over the stained area.
8. Let the slides dry completely.
**Optional:** Drying the stained slides can be accelerated by airflow or by drying them in an inclined position.
**Note:** Fixed cell suspensions can be stored at −20°C in methanol-acetic acid fixative and remain stable for several months if properly sealed to prevent evaporation. Stained slides are kept at room temperature in the dark, preferably in slide boxes, and can be evaluated for several years. However, prolonged storage may cause staining fading, especially with Giemsa.

### Data acquisition and evaluation

This section describes how to perform automated microscopy analysis using the Metafer scanning system to identify and count DCss in 100 metaphases per sample.

**Note:** Manual scoring can be performed using conventional light microscopy if the laboratory has no automated scanning equipment.

**Figure 2.**
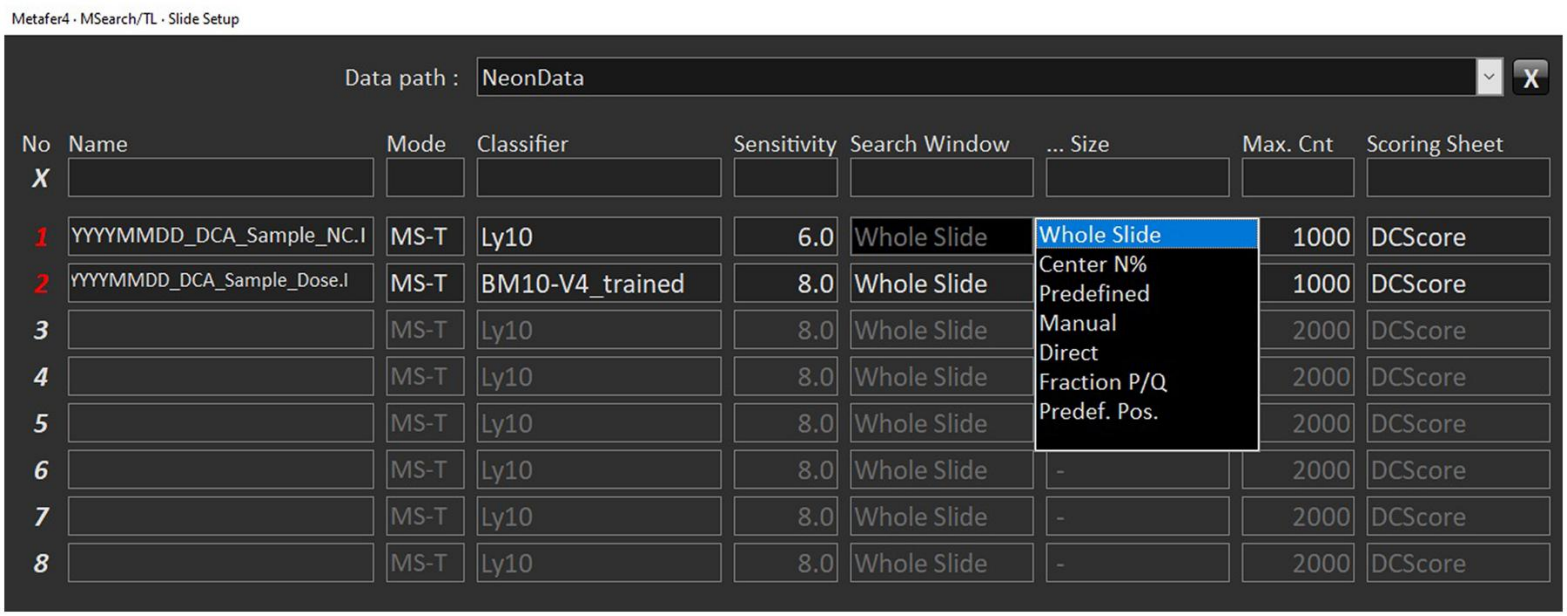
Menu for the slide setup. Recommended parameters for the first metaphase capture using MSearch/TL mode.

**Figure 3.**
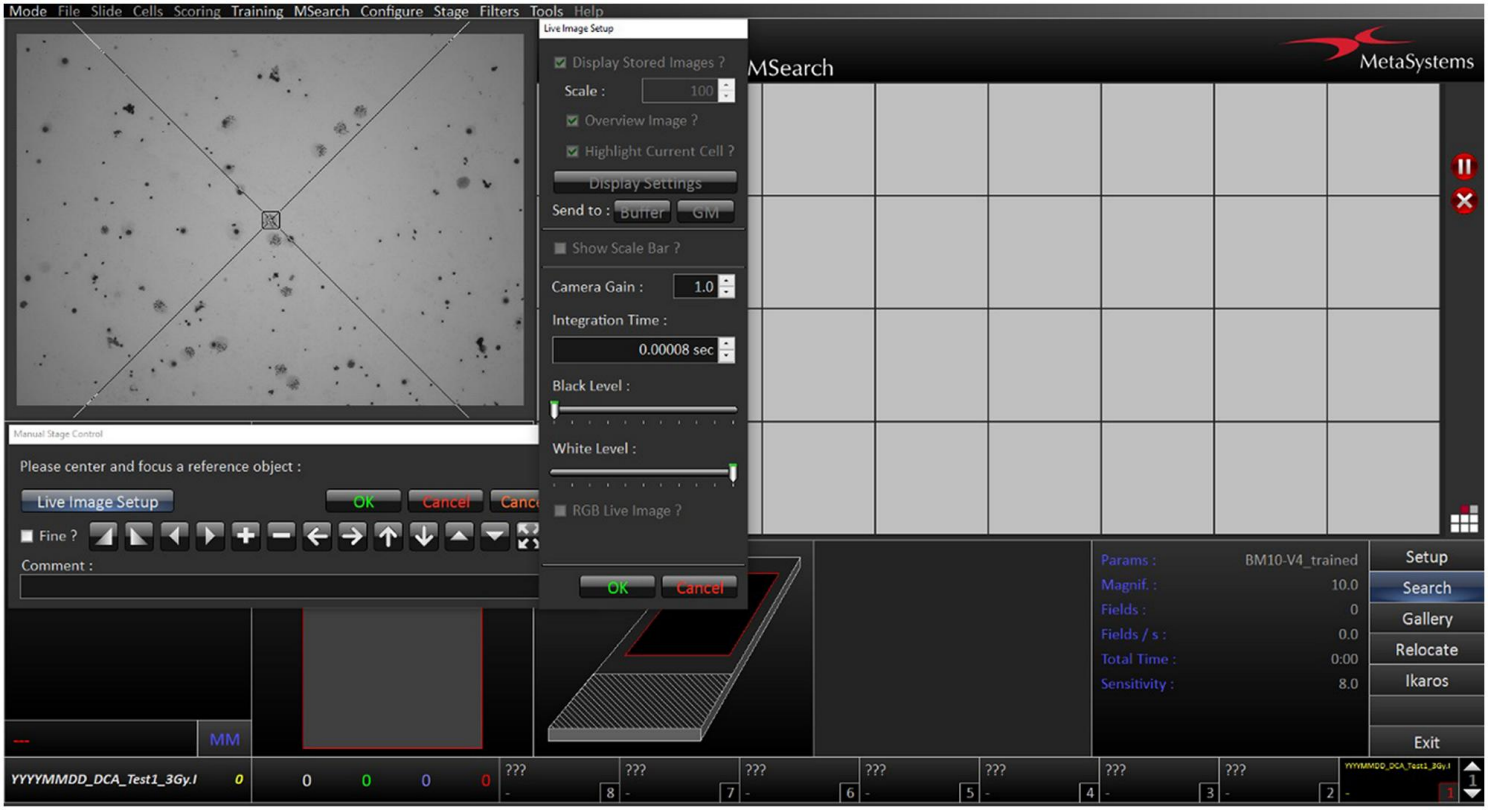
The focus position setup and the reference object selection (magnification 10x, Metafer 4 - MSearch).

**Figure 4.**
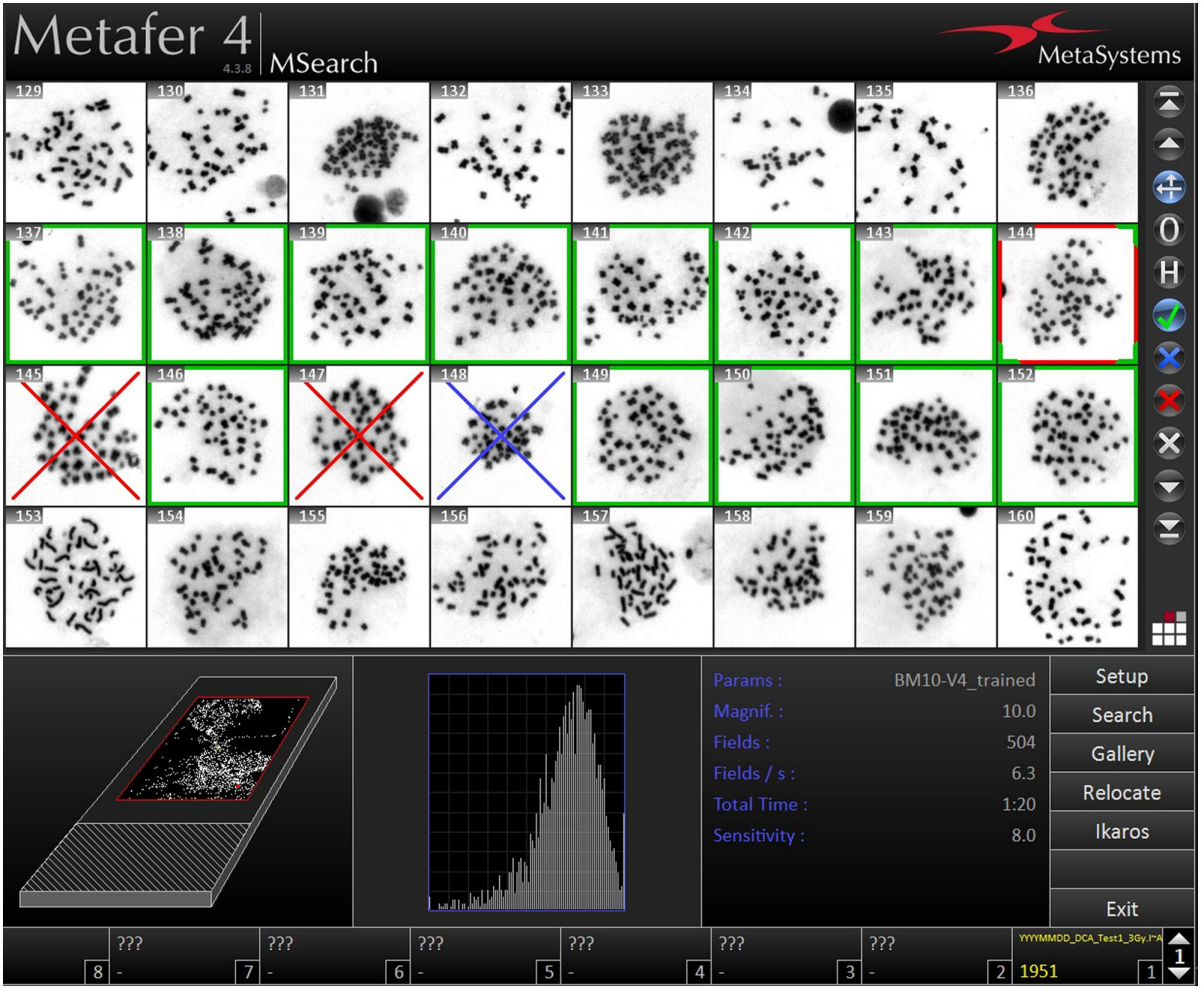
Sample metaphases selection meeting all parameters in analysis window (green frame—marked/accepted cell; red frame—active position; blue cross—rejected cell; and red cross—deleted cell).

**Figure 5.**
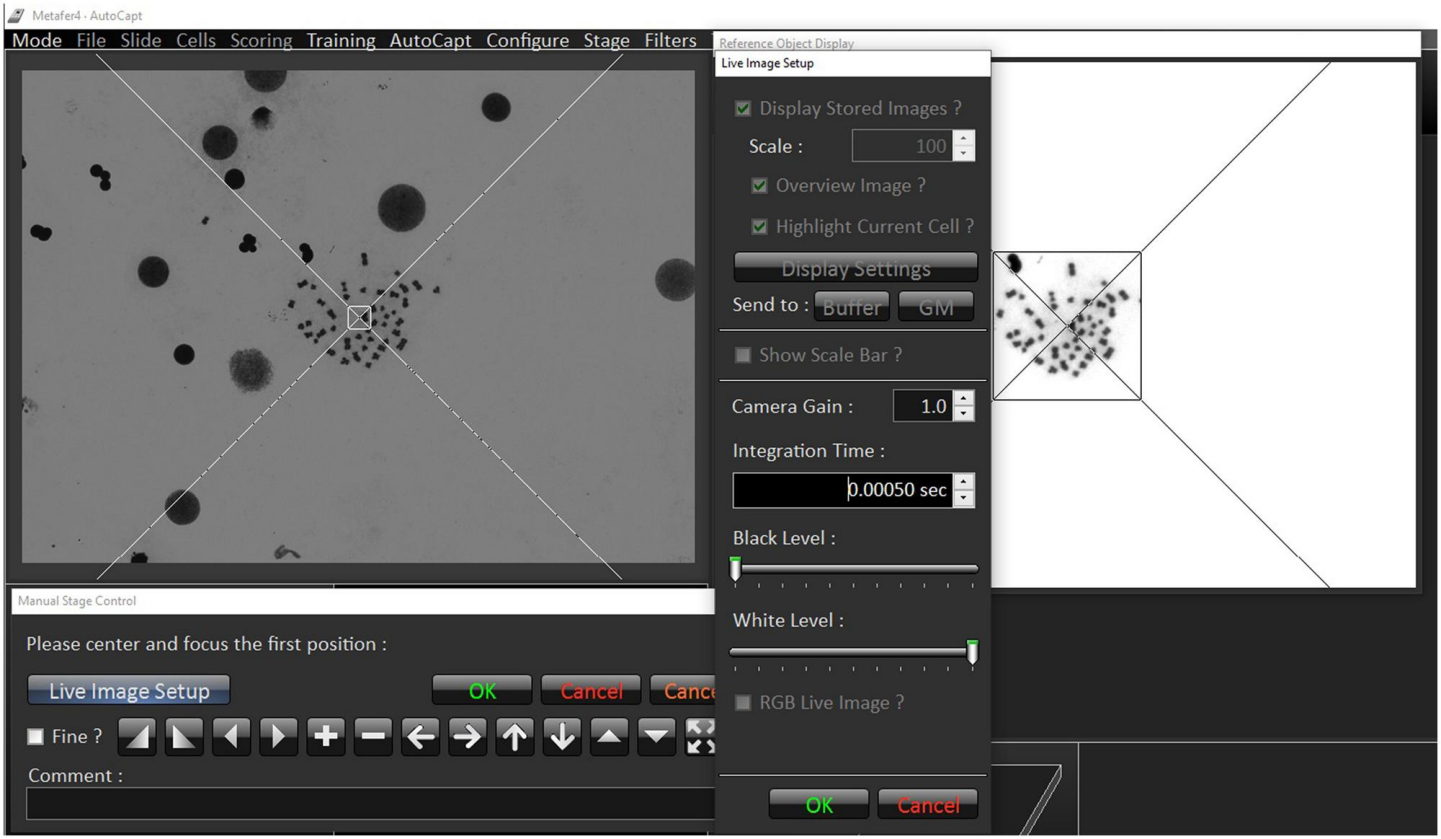
Focusing and centering of the first reference metaphase (magnification 63x, Metafer 4 - Autocapt).

**Figure 6.**
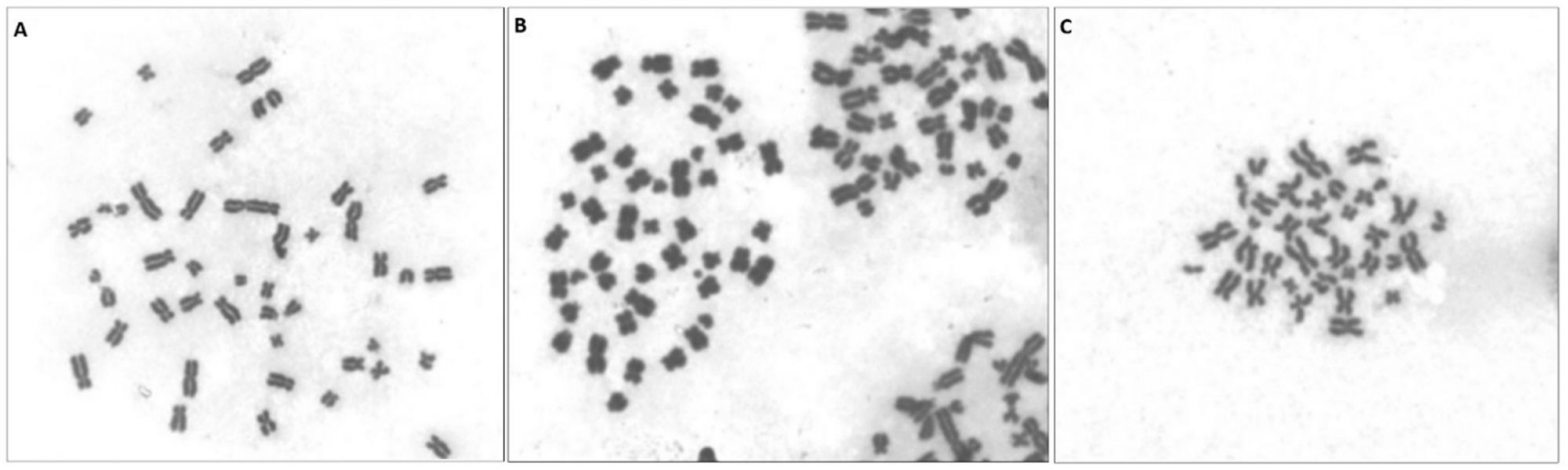
Metaphases inappropriate for evaluation: (A) metaphase with excessively spread chromosomes, (B) more than one captured metaphase, (C) a metaphase with an incomplete set of chromosomes.

1. Select only high-quality, high-resolution metaphases containing 46 chromosomes for analysis.
2. Count the number of DCs in metaphases meeting the criteria from the previous point. It is recommended that a minimum of 100 metaphases be evaluated per sample, and the experiment is repeated at least three times.
3. Conduct statistical comparisons between groups to identify significant differences. A *P* < 0.05 is considered statistically significant.
**Note:** The classifier is a predefined set of algorithmic parameters that guides the identification of target cells (e.g., metaphases) based on object features such as size, shape, contrast, texture, and staining intensity. These classifiers are either provided by MetaSystems or can be trained by experienced users using representative sets of image fields. Typical users do not have direct access to the internal classifier parameters. However, detection performance can be optimized by adjusting general settings such as detection sensitivity, applying quality filters (e.g. sharpness or area thresholds), or refining the classifier using MetaSystems tools. Therefore, adaptation may be needed to achieve optimal detection under current conditions.
4. Before using the automatic scanning system, clean the platform with an alcohol-based cleaner and dust it off. Place the slides in the holder on the moving platform. Ensure the slides are in the correct position and securely fixed with a clip to prevent them from falling out.
5. Select **MSearch** mode from the **Mode** menu and define parameters in **Setup** (Fig. 2).
6. Select the data path to determine where the image files from the search are stored. Name each scan uniquely. Choose the appropriate mode, **MSearch-Transmitted Light (MS-T)**, set a classifier and required sensitivity, select **Search Window**, and set the **Maximum Cell Count** to stop scanning once the desired number of cells is reached. Confirm settings by **OK**.
**Note**: It is necessary to select the available preset classifiers for lymphocytes or create and use a trained classifier based on your training data. For metaphase search, a sensitivity of 6 to 8 is optimal. It is recommended that the search window be set to scan the whole slide.
7. Open the dialogue by clicking the **Search** button in the side bar and select a reference point using the 10x objective.
**Note:** The microscope objectives change automatically according to the set classifier.
8. Adjust the lamp intensity manually using the rotary selector under the microscope display, or adjust the integration time in the **Live Image Setup**, and adjust the condenser aperture (50–60%).
9. Use the trackball to locate, center, and focus the object manually. Confirm the reference object by clicking the left mouse button (Fig. 3), then click **OK.** The system will automatically move to the next position on the scanning platform. Repeat this process for all slides.
10. After final settings control and confirmation, the system automatically adjusts the focus and defines the field in a dedicated slide area. In the lower right window, you will find information about parameters, the number of fields, and scanning time.
**Note:** Once the scan is complete, the data is automatically saved, and the analysis window displays the resulting metaphase scans and their total number.
11. In the analysis window, mark the selected metaphases (Fig. 4) that match the evaluation parameters and meet all criteria for the next step. Only the marked objects can be submitted for further scanning under higher magnification.
12. Select **AutoCapt** mode from the **Mode** menu and click **Setup** in the sidebar.
13. Mark the positions of the slides you want to scan. Active slides are highlighted in red.
**Note:** Do not rename or change the sample name at this point. The system will mark each subsequent scan with a letter automatically.
14. Set the mode **AutoCapt**, classifier **CaptMetaTL**, or any other optional classifier, and confirm by clicking **OK**. There is no need to set additional parameters.
15. Manually use the buttons on the right side of the microscope to lower the stage and drop immersion oil onto the slides without changing the position of the slides. Return the stage to the default position and click **Search.** The oil objective (63x) is changed automatically.
16. Adjust the condenser aperture (80%).
17. The user is prompted to adjust the focus start position again. A reference object is selected automatically, and the software prompts the user to focus and center the first marked reference metaphase for each slide and to confirm settings with **OK** (Fig. 5).
18. Confirm the system message and click **OK** to start scanning the selected objects under higher magnification (63x). The lower right window displays information about parameters, the number of objects, and scanning time. When the scan is complete, the data is automatically saved, and the analysis window displays the resulting metaphase scans and their total number.
19. When the scan is complete, perform a final quality check of the metaphases in the analysis window, following the same procedure as after the first scan.
20. Mark, reject, or delete the objects you want to analyse, edit, or advance for further manual analysis, and select **Gallery/Relocate** in the sidebar.
21. The **Gallery** displays small images, including labels and order.
22. In **Relocate**, metaphases are displayed at their captured size, individually in order, including information about Cell number and Cell ID, parameters, and quality. Objects, chromosomes, DCs, fragments, and other aberrations can be counted in this display mode.
**Critical:** The final selection and evaluation of metaphases is an important step. Evaluating metaphases not meeting the criteria (Fig. 6) can lead to false positives and skewed results.

## Expected outcomes

Since the presence of DCs is always lethal for the cell, it is crucial for the survival and recovery of the organism after irradiation that the number of cells with DCs is kept as low as possible [13]. Substances that can reduce the frequency of DCs after exposure to IR can be considered radioprotective [7, 12]. Therefore, in addition to its established use in biodosimetry, the DCA is also a suitable method for determining the radioprotective potential of substances [10, 11].

The assessment of cell division is an important aspect of quality control in DCA. Monitoring cell proliferation during DCA helps identify potential issues related to culture conditions or cytotoxic effects of tested substances. The cell division rate is typically assessed by estimating the mitotic index—the percentage of cells in mitosis at harvest time. To do this, examine several microscope fields and count the number of mitotic cells relative to the total number of cells observed to estimate the proportion of dividing cells. The metaphase yield per slide in DCA can also be a practical and indirect indicator of cell proliferation. Consistently low metaphase yield may reflect poor culture conditions, cytotoxic effects of tested substances, or insufficient mitotic activity. Monitoring these parameters helps verify culture success and supports troubleshooting when necessary.

The present protocol aims to evaluate the protective efficacy of piperazine derivatives, amifostine, and its active metabolite WR-1065 against the formation of radiation-induced DC aberrations in cultured human peripheral blood lymphocytes. For the successful execution of the test and subsequent evaluation, it is essential to pay attention to critical points and notes, particularly adhering to appropriate conditions during culture, optimal ratios of culture media and supplements, concentrations of mitogenic agents, and the timing and adherence to procedures for fixation and preparation of slides. Evaluator expertise is crucial for accurate assessment, including selecting high-quality M1 metaphases and the distribution of chromosomes to facilitate easier and more precise identification.

**Figure 7.**
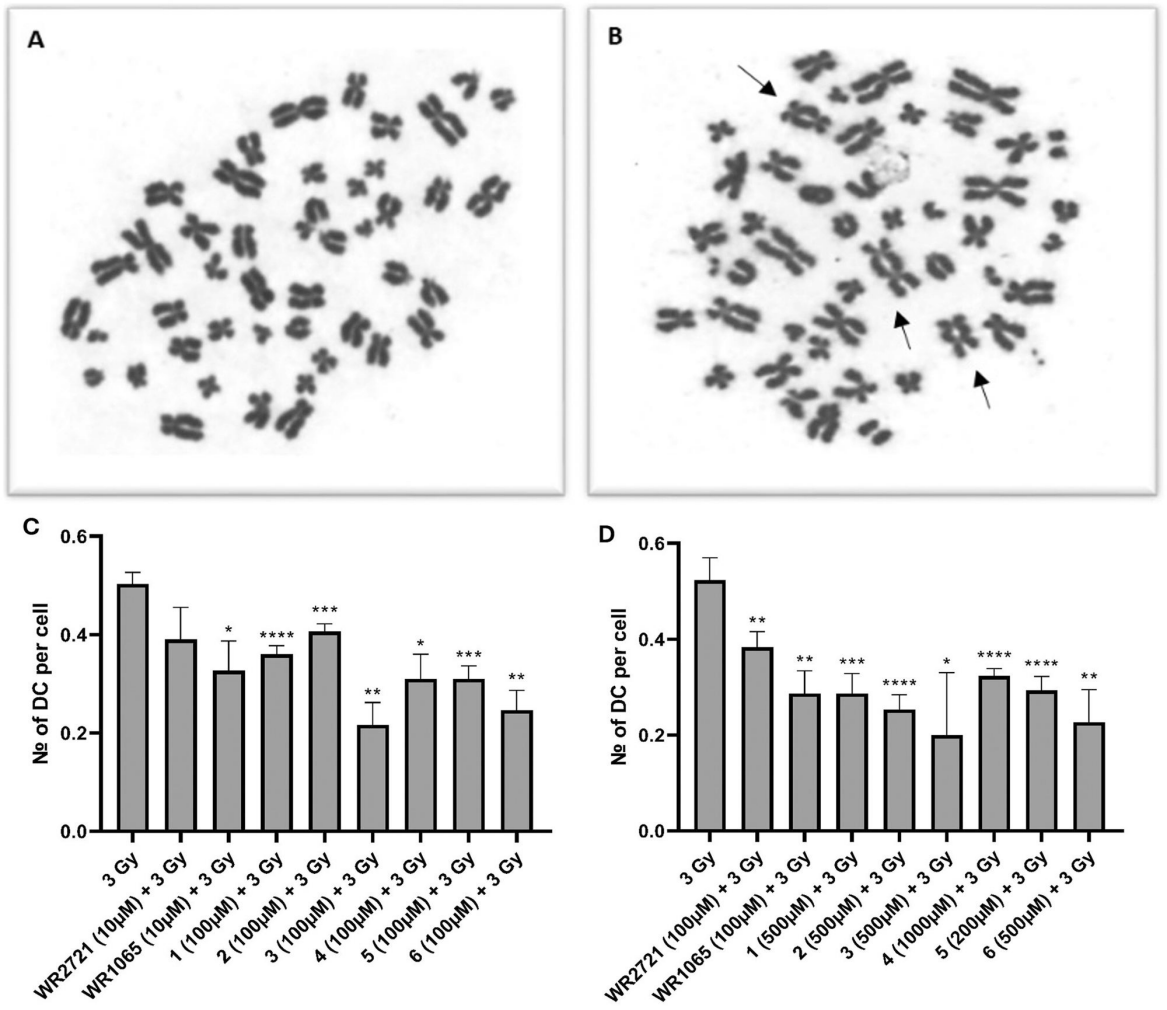
Example of metaphase in a non-irradiated control sample without DCs (A), metaphase after 3 Gy irradiation with DCs (B); and graphical representation of the number of dicentric chromosomes (DC) per cell after exposure to IR (3 Gy) alone or after 1-h pretreatment with the compounds at optimal non-toxic concentration [C] and maximum tolerated concentration [D]. The incubation interval was 48 h, and then the cells were subjected to DCA. Error bars represent the standard deviation. The asterisks above the individual bars represent statistical significance (*P < .05, **P < .01, ***P < .001, ****P < .0001) related to 3 Gy. Reproduced from Chmil *et al*. 2024 [7] with permission from the Royal Society of Chemistry under CC BY 3.0.

Results from our study suggest that the frequency of DCs in the control group will be <1 dicentric per 1000 cells, consistent with baseline levels of chromosomal aberrations in the absence of radiation exposure. In contrast, the irradiated-only group is anticipated to show a significant increase in DC formation, reflecting the cytogenetic damage caused by IR. Pre-treatment with radioprotective agents is expected to reduce the frequency of DCs compared to the irradiated-only group, thereby demonstrating their effectiveness in mitigating radiation-induced chromosomal damage (Fig. 7). A comparative analysis further elucidated which tested compounds provided superior radioprotection and whether the novel piperazine derivatives outperformed the established radioprotectant amifostine in their effects. Compounds 3 and 6 demonstrated the best results, suggesting that the piperazine derivatives are more effective than amifostine. This will contribute to optimizing protective strategies in radiotherapy and other radiation-related applications.

From the data of the irradiated-only group, interindividual differences in the DCs per cell ratio were calculated. The results showed an average of 0.514 DCs per cell, a standard deviation of 0.035, and a coefficient of variation of 6.890%, indicating some variability across 12 different donors. The overall variation is relatively low, suggesting good consistency among the donors’ responses. The standard deviation further supports this observation, indicating that the number of DCs per cell does not deviate significantly from the average value.

Compared to other methods for evaluating radioprotective effects, such as the micronucleus assay, which is faster and simpler but lacks specificity, the DCA offers a direct and precise correlation with radiation dose, serving as a highly accurate biomarker for IR exposure. While the chromosomal aberration assay provides a broader spectrum of detectable structural chromosomal changes, including translocations and deletions, DCA remains the preferred standard due to its targeted and reliable detection of radiation-induced double-strand breaks, specifically leading to DCs formation, which is an established indicator of radiation exposure [9]. On the other hand, the clonogenic assay, which assesses overall cell survival and proliferative capacity following radiation exposure, does not offer the chromosomal specificity inherent to DCA. Similarly, although the comet assay effectively detects DNA strand breaks at the single-cell level, it does not differentiate between various types of chromosomal damage as comprehensively as DCA. Despite being more labor-intensive and requiring a higher level of technical expertise, DCA's ability to accurately correlate DC frequency with radiation dose makes it an essential tool in radiobiology and biodosimetry, providing superior accuracy in assessing radiation-induced chromosomal damage compared to other available methods [10, 11].

## Quantification and statistical analysis

The result of DCA is expressed as the total number of DCs related to the number of inspected metaphase cells. Thus, it represents the average number of DCs per cell in a given sample. At higher doses of radiation, tricentric and, rarely, tetracentric chromosomes may also appear. For the calculation, a tricentric chromosome is counted as 2 DCs, and a tetracentric chromosome is counted as 3 DCs [8].

It is recommended that the mitotic index be determined as part of the DCA. The mitotic index is a measure of cell proliferation and is essential in DCA to ensure that a sufficient number of cells have progressed through mitosis, which is necessary for accurate detection of DCs.

The RF was used to express the radioprotective effect of the tested substances. The RF is calculated as the ratio of the number of DCs per cell in the irradiated-only group to the number of DCs per cell in the treatment group (Equation (1)). The RF indicates how many times the number of DCs decreased in the sample compared to the irradiated-only group.


(1) $$RF=\frac{{\mathrm{number}\;of\;\mathrm{DCs}\;\mathrm{pe}r\;\mathrm{cell}}_{\mathrm{irradiated}-\mathrm{only}\;\mathrm{group}}}{{\mathrm{number}\;of\;\mathrm{DCs}\;\mathrm{per}\;\mathrm{cell}}_{\mathrm{treatment}\;\mathrm{group}}}$$

The statistical analysis and graph creation were performed using GraphPad Prism 8.4.3 software. A one-way analysis of variance (ANOVA) was used for data evaluation, specifically employing the Brown-Forsythe and Welch ANOVA tests. To compare statistically significant differences between the test and control samples, an unpaired t-test with Welch’s correction was utilized. The confidence level was set at 0.05 (95% confidence interval). The error bars on all graphs represent the standard deviation.

## Limitations

The DCA protocol has several limitations. DCA is typically performed on lymphocytes or other cell types responsive to mitogen stimulation. High-resolution microscopy and well-prepared metaphase spreads are essential. However, poor slide preparation or overlapping chromosomes can hinder accurate detection and scoring. Variability in cell culture conditions, such as mitogen concentrations and environmental factors, affects cell proliferation and metaphase quality, impacting result consistency and reliability. Additionally, technical variability, including differences in slide preparation, staining, and scoring performed by different evaluators, can lead to inconsistent results. The method is generally time-consuming and requires highly skilled personnel, careful planning, and effective resource management to ensure accurate and reliable outcomes.

## Troubleshooting

### Problem 1

Inconsistent conditions during lymphocyte cultivation, such as fluctuations in humidity, temperature, CO_2_ concentration, or imbalances in the culture medium’s composition, can negatively affect cell proliferation and the yield of cells in metaphase. Variations in these parameters may lead to suboptimal cell division, resulting in insufficient metaphase cells for DCA.

### Potential solution

- To ensure optimal cell growth and division throughout the entire culture period, it is crucial to maintain precise conditions of humidity, temperature, and CO_2_ concentration. The culture medium should also have a balanced FBS, glucose, and glutamine composition.
- To promote better cell proliferation and ensure an adequate number of cells in metaphase, using a medium with a higher glucose content (up to 2 g/L, RPMI 1640 Medium [ATCC modification], Gibco) and/or stable glutamine (GlutaMAX™) can be beneficial. Maintaining these conditions consistently will help achieve reliable results in DCA.

### Problem 2

During the cell fixation and slide preparation for DCA, several issues can arise that may compromise the quality of the preparations. Inadequate fixation, improper cell handling, or suboptimal slide preparation techniques can lead to poorly spread metaphase chromosomes, clumping of cells, cytoplasmic debris, dark streaks on the metaphase background, excessive debris on the slide, or loss of material. These issues can result in low-quality images, making it challenging to score DCs accurately and potentially leading to unreliable results.

### Potential solution

- Constant, thorough, and slow mixing of the sample throughout the fixation process is necessary to prevent cell clumping, overlapping chromosomes, and material loss, resulting in better-preserved metaphase spreads.
- The remaining supernatant after the final fixation step affects the final cell density on the slide. The remaining suspension can be further concentrated or diluted with fixative to achieve the desired cell density if needed.
- Before applying the cell suspension to the slides, ensure the cells are thoroughly suspended in the remaining liquid.
- Shake off the excess liquid from the slides just before adding the cell suspension to ensure better distribution of chromosomes.
- Use cold glass surfaces to aid in the proper spreading of metaphases, and carefully control the evaporation rate of the fixative based on room temperature and humidity.
- If issues with the quality of metaphase spreads persist, consider adjusting the ratio of cell fixation solution components or repeating the fixation step. After the initial fixation, you can store the cells in the freezer for a few minutes and then repeat the fixation with freshly prepared, ice-cold fixative. This additional step can improve cell preservation and enhance the overall quality of the metaphase spreads.

### Problem 3

During the lymphocytes cultivation for DCA, variations in timing, PHA, and colcemid concentrations can significantly affect the accuracy and consistency of results. Extended cultivation time may cause cells to progress beyond metaphase or suffer increased chromosomal damage, potentially leading to inaccurate DC frequencies. If PHA concentration is too low, lymphocyte division may be insufficient, resulting in fewer metaphase cells and reduced assay reliability. Conversely, excessively high mitogen levels can cause cell clumping, poor metaphase spreads, and artifacts, hampering the scoring of DCs. Insufficient colcemid concentration decreases the yield of metaphases, while excessive concentrations or early addition can be toxic to cells, causing undue cell contraction and further complicating the analysis.

### Potential solution

- To optimize the cultivation process, it is recommended to standardize the cultivation duration and introduce colcemid 2–3 h before harvest. This approach ensures cells are arrested in metaphase, enhancing the accuracy of cytogenetic analysis. A cultivation time of 48–52 h is generally recommended, with adjustments based on the observed cell proliferation rates. When using short-time colcemid treatment to arrest cells in metaphase, verifying the predominance of first-division metaphases (M1) over second-division metaphases (M2) can be important, especially if the metaphase yield is suboptimal.
- Conduct a titration experiment to determine the optimal concentration of mitogen that stimulates adequate cell division without causing clumping or artifacts. PHA is typically used in the culture medium at a concentration of 2%, but the optimal concentration should be validated based on specific experimental conditions. If necessary, the concentration can be gradually increased to 5% to enhance cell division, ensuring an adequate number of metaphase cells for accurate cytogenetic analysis.
- Implement a routine monitoring protocol to assess the quality of metaphase spreads during the culture process. This can include regular checks on cell division rates and preliminary analysis of metaphase spreads to ensure they meet the required standards before proceeding with full-scale DCA.

## Conclusion

This protocol presents a comprehensive, step-by-step procedure designed to assess the radioprotective effects of various substances using the DCA. The DCA is a highly specific and sensitive method for detecting radiation-induced chromosomal aberrations, making it an essential tool in radiation biology and biodosimetry.

The protocol begins with the preparation of human peripheral blood lymphocytes and their treatment with potential radioprotective agents. It describes the exposure to IR, followed by culturing, harvesting, and staining the cells for microscopic analysis. The identification and scoring of DCs are key components of the assay, ensuring accurate quantification of radiation-induced damage and assessing the tested substances’ protective effects. The key procedures are highlighted as critical steps.

By providing a comprehensive guide to the DCA, this protocol facilitates the standardized evaluation of radioprotective compounds. It ensures reproducibility and accuracy in detecting DCs, thus enabling researchers to compare the efficacy of different agents reliably. Integrating both automatic data acquisition and manual analysis is highlighted, offering flexibility and enhancing the robustness of the assay.

This standardized approach is crucial for advancing the field of radioprotection, as it allows for consistent and comparable results across different studies and laboratories. The protocol is a valuable resource for researchers aiming to develop and evaluate new radioprotective agents, ultimately contributing to improved radiation countermeasures in medical, industrial, and emergency settings involving IR.

Future research should focus on refining the assay conditions, exploring new potential radioprotective substances, and incorporating advanced imaging and analysis technologies. By adhering to this detailed protocol, researchers can ensure the reliability and validity of their findings, thereby advancing our strategies for developing and assessing radioprotective agents.

## Data Availability

All underlying data for this article are available at 10.1039/d4md00311j.
